# Improving Hurricane Preparedness Among Galveston County Older Adults: Description of a Successful Community-Based Quality Improvement Model

**DOI:** 10.1177/23337214241278550

**Published:** 2024-09-21

**Authors:** Jennifer Young, Jessica den Herder, Tammie Michael, Diana P. Bernardez, Zachary Carson, Mukaila Raji, Yong Fang Kuo

**Affiliations:** 1 The University of Texas Medical Branch, Galveston, USA

**Keywords:** gerontology, nonpharmacological/holistic interventions, nursing, population health, quality improvement

## Abstract

Older adults are at high risk of experiencing injury, exacerbations of their chronic conditions, and death when evacuations are ordered because of hurricanes or natural disasters. The Homebound seniors residing in Galveston County are at a particularly high risk of morbidity and mortality during evacuations for hurricanes. This paper described the impact of a quality improvement intervention designed and implemented by nurse practitioners during the 2022 and 2023 hurricane season. The education program aimed at increasing the hurricane preparedness of the home-bound patients. Of these patients, 190 returned pre and post surveys. Interventions showed a 43% increase in patients having an evacuation plan in the event of a hurricane, 633% increase in STEAR registration, 16% increase in patients having access to emergency supplies, and 34% increase in patients having an emergency contact list with up-to-date medication list. All improvements on hurricane preparedness items were significant (*p* < .0001) except for the need for assistance in case of an evacuation. Our findings suggest a need for continuous hurricane preparedness education in the community to ensure safe evacuation, improve hurricane preparation, and increase the number of seniors who registered with the State of Texas Emergency Assistance Registry.

## Introduction and Background

The National Oceanic and Atmospheric Administration (2022) stated the average Atlantic hurricane season has 14 named storms, 7 hurricanes, and 3 major hurricanes of category strengths 3, 4, or 5 on the Saffir-Simpson Hurricane Wind Scale between years 1991 and 2020 (U.S. Department of Commerce/NOAA, 2022). Studies completed after Hurricanes Irma and Harvey have shown higher rates of mortality in Gulf Coast counties that experienced a hurricane disaster (Bell et al., 2023). According to National Institutes of Health (NIH), it was determined that the stress related to hurricane evacuation in older adults placed them at a higher risk of inhibiting their activities of daily functioning up to 6 years after the hurricane happened (Bell et al., 2023). Homebound seniors residing in Galveston County with proximity to the Gulf of Mexico are at higher risk for morbidity and mortality associated with severe natural disasters such as hurricanes. The State of Texas Emergency Assistance Registry (STEAR) provides local emergency planners with information for citizens who may require emergency assistance during a disaster, such as help with evacuation before and after a hurricane. STEAR registration must be completed annually. In April 2022, the Galveston County Office of Emergency Management (OEM) reported only 33 residents registered with STEAR of an estimated 6,509 homebound persons over 65 years of age in Galveston County per US Census 2022 data (U.S. Census Bureau, 2020, 2022). Al-Rousan et al. (2015) surveyed 1,304 adults ages 50 years or older and found only 23.6% had a specific plan for how to respond to a disaster such as a hurricane. Compared to prepared adults, the unprepared Alzheimer’s dementia and related dementia(ADRD) population of 85 years and older had the highest mortality rate associated with exposure to hurricanes, particularly Hurricane Harvey and Hurricane Irma. Mortality rates peaked for the vulnerable ADRD population “3 to 6 months after Hurricanes Irma and Harvey landfall but not after Hurricane Florence” ranging from 10.9% for Harvey and 6.2% for Irma (Bell et al., 2023). The US Department of Veterans Affairs (VA) examined the impact of disasters on older adults and found older adults at increased risk for post-traumatic psychopathology (U.S. Department of Veterans Affairs, 2023). The VA cited providing education to older adults on community resources could be helpful.

Primary care nurse practitioners identified the need for more education on hurricane preparation and evacuation planning among homebound older adults in Galveston County. This geriatric homebound primary care program—led by four nurse practitioners and three physicians—provide primary care to over 400 homebound older adults within Galveston County. The objective of the current study was to determine whether the hurricane preparation-specific educational interventions provided by geriatric homebound primary care team affect the baseline preparedness level of the Galveston County STEAR registrants. The education program on hurricane preparation was designed as a quality improvement intervention to increase Galveston County STEAR registrants’ level of hurricane preparedness by 20%, with a focus on the 2022 and 2023 hurricane season that falls between June 1^ST^ and November 30^TH^ of those years.

## Methods

The implemented quality improvement initiative provided education on hurricane preparedness to senior community centers and homebound seniors during geriatric home-based primary care visits. In 2022, education was provided to 115 homebound adults and 118 elderly adults at three senior community centers and one community church from May 2022 through September 2022. In 2023, education was provided to 150 homebound adults and 185 older adults at three seniors community centers, one adult day care center, and four community churches from June 2023 through September 2023. At the end of hurricane seasons of 2022 through 2023, 194 homebound seniors were surveyed about hurricane evacuation planning. The need for assistance during an evacuation was evaluated along with registration status for STEAR, emergency supplies on hand, and access to an emergency contact and medication list. Educational events and starter hurricane emergency supply kits were provided in 2022 and 2023 through fundraising over 4,000 dollars by the home-based primary care nurse practitioners and a 10,000-dollar grant.

Education focused on disaster and evacuation planning, the STEAR registration process, supplies needed to shelter in place, medication and durable medical equipment needs, disaster supply kit necessities, emergency contact with medication list, and use of identification bands with the information to include on it. Hurricane disaster preparation starter kits were supplied with the education and included a waterproof document bag, identification band, sharpie, dry goods, hand sanitizer, personal cleansing wipes, flashlights with batteries, first aid kits, STEAR registration forms, and Texas Ready education forms with checklists. Assistance was offered to families and older adults with registering for STEAR. Surveys were obtained during home visits with homebound geriatric patients for pre and post education analysis.

McNemar test—a non-parametric statistical method—was used for analysis of significant differences in improvement on the survey items by comparing the pre and post survey item responses of hurricane preparedness measures among the 190 participants who returned the surveys.

## Results

**Figure 1.**
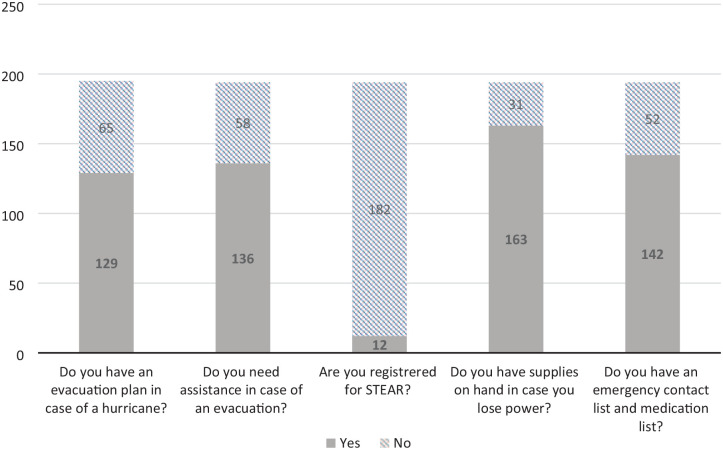
Pre-survey 2022.

**Figure 2.**
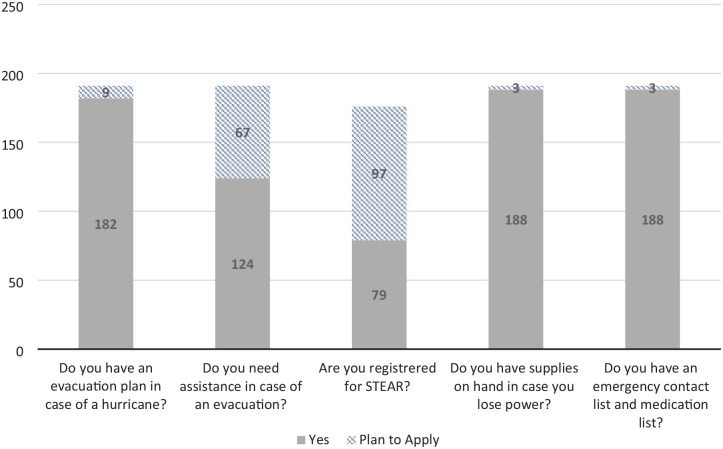
Post-survey 2022.

Table 1 showed that 194 home bound older adults were surveyed from 2022 to 2023 during routine home visits by the home-based primary care team. Table 1 showed that the median age of participants was 84 with a range of 67 to 103. Participants were predominately female (69%). Survey results revealed a 43% increase in patients having an evacuation plan in the event of a hurricane, 633% increase in STEAR registration, 16% increase in patients having access to emergency supplies, and 34% increase in patients having an emergency contact list with an up-to-date medication list. (Figures 1 and 2). Table 2 showed that among the 190 patients returned both pre and post surveys, the improvements on the hurricane preparedness items were significant (*p* < .0001) based on McNemar test. The change on the need for assistance in case of an evacuation was not significant.

**Table 1.** Demographics.

Total	194
Gender	
Female	135
Male	59
Ethnicity	
African American	62
Caucasian	99
Hispanic	27
Pacific Islander	6
Median age	84

**Table 2.** Survey Results.

Questions	No change	Yes to no	No to yes/plan to	*p*-Value
Do you have an evacuation plan in case of a hurricane?	136	0	54	<.0001
Do you need assistance in case of an evacuation?	166	17	7	.0639
Are you registered for STEAR?	109	0	81	<.0001
Do you have supplies on hand in case you lose power?	162	0	28	<.0001
Do you have an emergency contact list and medication list?	145	0	45	<.0001

**Figure 3.**
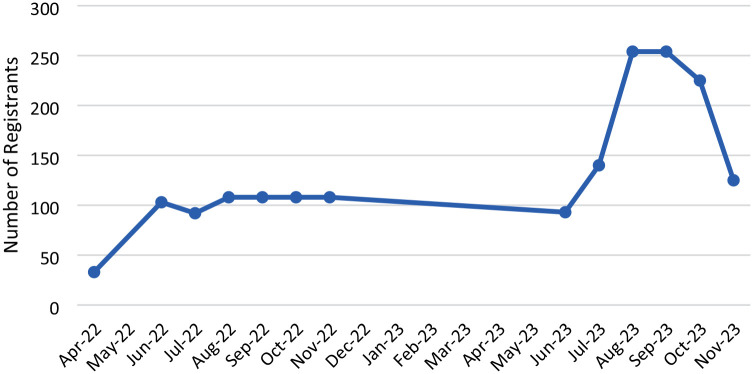
2022 to 2023 Galveston County, Texas S.T.E.A.R. Registrants.

Galveston County STEAR registration census was reported by the Galveston County office of emergency management and updated during hurricane season June 1st to November 30th. STEAR registration must be updated annually. In April 2022, Galveston County STEAR registrants totaled 33. In November 2022, Galveston County STEAR registrants totaled 108 resulting in a 272% increase. In November 2023, Galveston County STEAR registrants totaled 125, resulting in a 34% increase from April 2023 to November 2023. Overall increase of 278% in STEAR registrants since the start of the project in April 2022 to November 2023 (Figure 3).

## Discussion

The project to increase Galveston County STEAR registrants by 20% annually was successful with an initial 227% increase in 2022 hurricane season and 34% increase in 2023 hurricane season. The need for continuous education in the community for STEAR and hurricane preparation is critical for safe evacuation and hurricane preparation for the older adult community. The maximum recorded STEAR registrants during the 2022 to 2023 review period was 254 and ended with 125 registrants. Annual assessment for the need and renewal of STEAR assistance by homebound older adults can help the office of emergency management better plan for community needs. Among the 190 patients returned both pre and post surveys, the improvement on the hurricane preparedness items were significant. However, our intervention had no significant effect on the need of assistance in case of an evacuation.

## Limitations and Future Directions

Pre and post surveys to in-home education participants did not evaluate stress levels related to hurricane preparation. There is an opportunity for future studies to investigate the relation between stress and the knowledge base of resources available for disaster preparedness. Many individuals and community groups who received hurricane preparation education in 2022 requested repeat education in 2023 and assistance with renewal of their STEAR registry. Post survey results demonstrated statistically significant increases in hurricane evacuation planning, emergency supplies, STEAR registration, emergency contact list, and medication list. Galveston County did not experience a hurricane during the quality improvement project April 2022 to November 2023 which limited our project in monitoring for effectiveness of mortality interventions. Most of the homebound older adults who received hurricane preparation education in home had family support. Our study did not account for family support in the data collection. There is potential for further research of the role of family presence and support in relation to hurricane preparation. This quality improvement project to increase education on hurricane preparedness was limited to older adults attending local county community center older adult activity programs, local church older adult activity programs, an adult day care, and homebound older adults receiving primary care through home based primary care program. Partnerships with adult protective services, meals on wheels, home health companies, hospice companies, and other in-home primary care provider services would further expand the knowledge gap among homebound seniors.
